# The Implications of Bone Marrow Adipose Tissue on Inflammaging

**DOI:** 10.3389/fendo.2022.853765

**Published:** 2022-03-11

**Authors:** Nicole Aaron, Samantha Costa, Clifford J. Rosen, Li Qiang

**Affiliations:** ^1^ Naomi Berrie Diabetes Center, Columbia University, New York, NY, United States; ^2^ Department of Pharmacology, Columbia University, New York, NY, United States; ^3^ Center for Clinical and Translational Research, Maine Medical Center Research Institute, Scarborough, ME, United States; ^4^ Graduate School of Biomedical Science and Engineering, University of Maine, Orono, ME, United States; ^5^ Department of Pathology, Columbia University, New York, NY, United States

**Keywords:** inflammation, bone marrow adipocytes, inflammaging, aging, bone marrow adipose tissue (BMAT)

## Abstract

Once considered an inert filler of the bone cavity, bone marrow adipose tissue (BMAT) is now regarded as a metabolically active organ that plays versatile roles in endocrine function, hematopoiesis, bone homeostasis and metabolism, and, potentially, energy conservation. While the regulation of BMAT is inadequately understood, it is recognized as a unique and dynamic fat depot that is distinct from peripheral fat. As we age, bone marrow adipocytes (BMAds) accumulate throughout the bone marrow (BM) milieu to influence the microenvironment. This process is conceivably signaled by the secretion of adipocyte-derived factors including pro-inflammatory cytokines and adipokines. Adipokines participate in the development of a chronic state of low-grade systemic inflammation (inflammaging), which trigger changes in the immune system that are characterized by declining fidelity and efficiency and cause an imbalance between pro-inflammatory and anti-inflammatory networks. In this review, we discuss the local effects of BMAT on bone homeostasis and the hematopoietic niche, age-related inflammatory changes associated with BMAT accrual, and the downstream effect on endocrine function, energy expenditure, and metabolism. Furthermore, we address therapeutic strategies to prevent BMAT accumulation and associated dysfunction during aging. In sum, BMAT is emerging as a critical player in aging and its explicit characterization still requires further research.

## Introduction

All tissues are affected by aging, but diseases that weaken the skeleton constitute the most prevalent chronic impairments in the United States (1–3). Skeletal diseases and related conditions are of grave concern among the aging population as they have the potential to significantly compromise systemic and local functions and diminish quality of life. The increase in bone marrow adiposity (BMA) over a lifetime is thought to be a major contributor to age-associated chronic conditions such as osteoporosis, osteoarthritis, and cancer (4–7). Qualitative studies have reported changes in the bone marrow (BM) of humans since 1882 when Ernest Neumann recognized aging resulted in trabecular bone loss and most of the BM consisted of adipose tissue (8). Since then, studies in both rodents and humans have validated that aging is associated with a significant increase in bone marrow adipose tissue (BMAT) (9, 10) with a concurrent decline in bone mineral density (11). Over the years, considerable advancements have been made related to BM imaging and BMAT quantification in humans and rodents. In humans, quantitative magnetic resonance imaging (MRI) and spectroscopy (MRS) allows for noninvasive monitoring of BMAT development and expansion (12–15). This compares to osmium tetroxide and contrast enhanced computed tomography, considered the gold standard in rodents, which provides both volumetric and spatial quantification of BMAT (16). Notwithstanding the advances in methodologies, BMAT represents an understudied aspect of adipocyte biology. Distinct from peripheral adipose tissue, BMAT displays a unique response to physiological changes (i.e., aging, exercise, cold exposure, nutritional variations like high-fat diet and fasting) (17–20). Furthermore, given its unique location, BMAT directly influences mechanisms of bone remodeling, hematopoiesis, and inflammation within the BM microenvironment (21, 22).

In general, aging is associated with impaired tissue regeneration that is congruent with increased BMA and an inflammaging phenotype. Inflammaging is characterized by unresolved and uncontrolled inflammation and a dysfunctional immune response that exacerbate the aging process and age-related chronic diseases (23, 24). Furthermore, this process is believed to exacerbate the decline in the regenerative capacity of the skeleton (25) by affecting bone marrow stromal cell (BMSC) proliferation, frequency, and fate determination (25). With recent evidence supporting BMAT as an endocrine and paracrine organ capable of local regulation of the BM microenvironment, it is important to further understand the relationship between bone marrow adipocytes (BMAds) and the observed inflammaging phenotype in aging.

## The Effects of BMA on Bone Marrow Stromal Cells and Hematopoietic Stem Cells

### BMA and BMSC Potential

**Figure 1:**
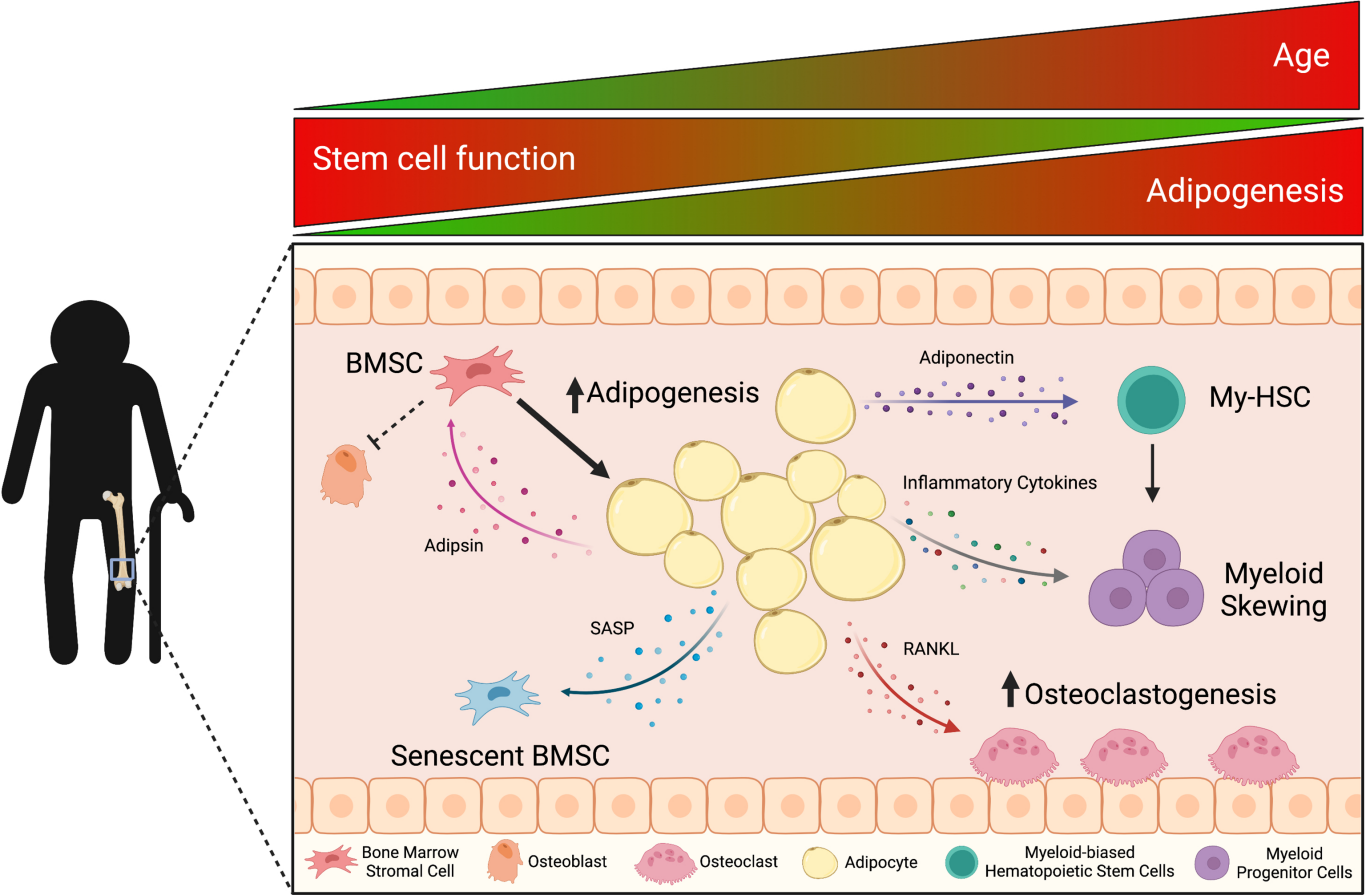
With age, the accumulation of bone marrow adipocyte (BMAd)-derived factors influences mechanisms of bone remodeling, hematopoiesis, and inflammation, which triggers a cascade effect within the bone marrow (BM) microenvironment. Aging is associated with increased bone marrow adiposity (BMA) and decreased bone mineral density. These classic characteristics of aging result from adipsin priming bone marrow stromal cells (BMSCs) towards adipogenesis and adipocytes (including pre-adipocytes in aged mice) secreting the pro-osteoclastic factor, RANKL. Adipocytes also secrete adiponectin and pro-inflammatory cytokines that skew hematopoietic stem cell differentiation towards the myeloid lineage, which is observed in the chronic inflammatory state of aging (inflammaging). In the BM, this pro-inflammatory microenvironment leads to senescence-associated secretory phenotype (SASP) factors decreasing BMSC potential and functionality. *This figure was created using BioRender.com*.

As we age, our capacity for tissue repair and regeneration in response to injury declines (**Figure 1**). Accordingly, bone repair is delayed and impaired as well. BMSCs are the foundation of bone regeneration by serving as the progenitor cells of osteoblasts as well as of adipocytes (26, 27). In addition, BMSCs support proliferation and differentiation of hematopoietic stem cells (HSCs), promote HSC engraftment in animal models, and can decrease inflammation under normal conditions (28). However, aging affects BMSCs through intrinsic and extrinsic factors. Intrinsically, BMSCs accumulate DNA damage, reactive oxygen species (ROS), and damaged proteins that may promote aging (29). Extrinsically, the composition of the BM niche and the growth factors and cytokines that are secreted into the local environment change with age (29) (**Table 1**). In particular, the increase in BMAds may disrupt the microenvironment structure and alter the fate of BMSCs. Age-related bone loss has thought to be driven in part by a decline in BMSC proliferation and function as well as increased commitment of BMSCs to adipogenic lineages (48). At the cellular level, the BMSC pool in the BM niche shows a biased differentiation towards adipogenesis at the cost of osteoblastogenesis in aging (48). Despite their regenerative capabilities, BMSCs were shown to have decreased differentiation potential when exposed to inflammatory environments (49). Josephson et al. revealed that skeletal stem/progenitor cell (SSPC) frequency significantly declined with increased age, and this directly correlated to a longer fracture healing time in a human cohort (25). Using *in vivo* and *in vitro* models, the authors recapitulated reduced bone healing commonly associated with advanced aging. SSPCs cultured with 52-week-old serum began to express pro-inflammatory cytokines (elevated IL-1a, TNF-a, RELA expression), illustrating the declined SSPC number and function were negatively affected by the cytokine milieu associated with age (25). The expansion of BMAT, which is known to actively produce pro-inflammatory factors, likely exacerbates this effect (45).

**Table 1:** BMAT-derived factors and the age-associated phenotype.

Age-Related Mechanism	Secreted Factors	Associated Effect	References
BMSC Potential	IL-1α	↓ skeletal stem/progenitor cell number and function	Josephson (25)
	TNF-α	↓ osteoblastogenesis	
	RELA		
Bone Loss	PPARγ	↑ adipogenesis	Fazeli (9)
	RANKL,	↓ osteoblastogenesis	Goto 2011 (55), Hardouin (30)
	Leptin, Resistin, Chemerin	↑ osteoclastogenesis	Hamrick (31), Thommesen (32), Han (33)
	Adipsin	-pro-inflammatory; regulates adipogenesis -prime BMSC differentiation towards adipogenesis	Aaron (19)
Hematopoietic Cells	Adiponectin	-prevents progenitor expansion	DiMascio (34), Naveiras (35)
		↑ myeloid skewing of HSCs	Pang (36), Ogawa (37)
		↓ BM cellularity	
Decreased Immune Fidelity	IL-6	-can alter immune response and hematopoiesis -inhibits B lymphopoiesis -induces the differentiation of immunoregulatory cells like regulatory T-cells and MDSCs -induces macrophage migration	Tanaka (38), Udagawa (39)
	IL-1, NLRP3		Kennedy (40)
	CCL2/MCP-1		Wang (41), Sinha (42),
	COX-2		Mahic (43), Obermajer (44)
Cellular Senescence	NF-kB (pro-inflammatory gene)	↑ pro-inflammatory cytokines	Miggitsch (45), Pangrazzi (23)
	IL-1α, IL-1β, TGF-β (pro-inflammatory cytokines)	↑ ROS	da Silva (46)
	p21, p16 (tumor suppressing genes)	↓ proliferative and differentiation capacities of surrounding cells	Josephson (25),
	CXCL1/2, CCL2/MCP-1 (chemokines)	↓ stem/progenitor cell number and functionality	Kovtonyuk (47)

↓ = down-regulates/decreases; ↑ = up-regulates/increases.

### Adipogenesis and Bone Loss

Aging studies have shown increased BMAT coincides with decreased bone mass, suggestive of a link between bone formation and BMA. The general understanding is a common progenitor cell undergoes adipogenesis at the expense of osteogenesis (27, 48, 50, 51). For example, it has been shown that upregulation of PPARγ promotes the differentiation of BMSCs into adipocytes while repressing osteoblast differentiation. In aging, the increased expression of PPARγ in the BM leads to enhanced adipogenesis and reduced osteogenesis (9). In addition to expression, post-translational modification of PPARγ, particularly acetylation, is also critical to this lineage determination (19, 52) thus, PPARγ is appreciated as a critical lineage-switching regulator. However, this bifurcated differentiation path between adipocytes and osteoblasts has remained poorly understood, despite the elucidation of PPARγ expression in the BM. Recent studies have delineated mesenchymal progenitors to their bi-lineage differentiation stages and characterized non-proliferative, adiponectin-expressing BMAd precursors, termed MALPs (marrow adipogenic lineage precursor) (53). These are thought to secrete a number of factors that can drive bone loss such as RANKL. Upon maturation, BMAT is responsible for the release of adipokines and free fatty acids that potentially interfere with bone formation (19, 52). For example, adipsin is among the group of adipokines released by BMAT expansion that has been shown to retroactively affect BMSC differentiation by priming these cells toward adipogenesis (19).

Coinciding with the increase in BMA, often an age-related decline in trabecular bone volume, but not in cortical bone, is observed (9). The impaired skeletal health with aging is accounted for not only by defective bone formation capabilities but also by accelerated bone resorption through increased osteoclast number and/or activity (54). In contrast to the repressive function on osteoblasts, BMAds play a favorable role on osteoclasts. Primary human femoral BMAds were shown to express the pro-osteoclastogenic factor, RANKL, and through direct cell contact mediate the differentiation of osteoclast precursors (30, 55, 56). In murine studies, an age-dependent increase in osteoclastogenesis was observed (57). Additionally, RANKL expression was shown to be associated with BMAd differentiation and with pre-adipocytes in the BM of aged mice (30, 58). This creates a self-reinforcing cycle of osteoclastogenesis and adipogenesis which leads to increased deleterious effects on the bone architecture and increases the incidences of fractures within the elderly (54). Furthermore, osteoblasts in aged mice (16 months old) were found to exhibit markedly impaired adhesion to the bone surface and significantly reduced mineralization (59). Thus, the age-associated decline in bone mass is an integrative pathology of BMAds filling the BM cavity and their crosstalk to bone remodeling cells.

### BMA and Hematopoietic Cells

While BMAds have a defined function as regulators of bone turnover, evidence also suggests BMAT impacts hematopoietic activity (45, 48). Human BMAds were reported to support differentiation of CD34^+^ HSCs into myeloid and lymphoid immune cells (60). Accordingly, myelopoiesis was shown to positively correlate with increased adipogenesis and reduced osteoblastogenesis in the senescence-accelerated mouse prone 6 (SAMP6) mouse model, representative of advanced aging (61). In diet-induced obese mice an enhancement in hematopoietic and lymphopoietic BM cell populations were correlated with increased marrow adiposity (62). In contrast, lipid-laden BMAds were linked to the suppression of growth and differentiation of HSCs (35, 63) and were considered negative regulators of the hematopoietic niche (64, 65). This suppressive activity was primarily attributed to reduced production of granulocyte-macrophage colony-stimulating factor (GM-CSF) and granulocyte colony-stimulating factor (G-CSF) as well as increased secretion of neuropilin and lipocalin-2 (35, 66, 67). Of note, BMAds are a significant source of plasma adiponectin in mice during calorie restriction and in cancer patients receiving radiotherapy or chemotherapy (20). Moreover, increased BMA during aging has been negatively correlated to hematopoietic cell function during aging through the secretion of adiponectin (20). Adiponectin appears to positively affect multipotent stem cells proliferation, but not more committed progenitor cells (34), a phenomenon suspected in preserving the HSC pool while preventing progenitor expansion (35). This ultimately highlights the anti-inflammatory properties of adiponectin (68) and the dynamic relationship between BMAds and the hematopoietic niche. Overall, aging in humans and mice, a process associated with increased BMA (69–71), induces myeloid skewing in HSCs (36), while promoting an overall decrease in BM cellularity (37).

## Age-Related BMAT Expansion Results in Decreased Immune Fidelity and Cellular Senescence

### Decreased Immune Fidelity

With aging, inflammaging is thought to be a major contributor to the decline in fidelity and efficiency of the immune system. The immune system waxes and wanes in response to stimuli. A decline in immunocompetency or the capacity for a normal functioning immune system with aging can increase susceptibility to infections, decrease the number of T- and B-cells as myelopoiesis occurs (the process in which innate immune cells develop from myeloid progenitor cells), and increase the prevalence of autoimmune diseases (47). Gasparrini et al. analyzed cytokines produced by BMAT and found 53 proteins upregulated in aging (72), one of which they identified as IL-6, a well-known pro-inflammatory protein that can affect immune response, hematopoiesis, and suppress bone formation (38, 39). *In vitro* cultures of BMAds were shown to secrete adipocyte-derived soluble factors that inhibit B lymphopoiesis, particularly at the earliest progenitor stage in which differentiation into pre-pro B-cells occurs, while simultaneously promoting the differentiation and subsequent proliferation of HSCs towards the myeloid lineage (73). In humans and mice (74–76), B lymphopoiesis wanes in mid (77) and late stages of life (73, 78, 79). In mice, the decline in B lymphopoiesis has been attributed to BMAds altering the BM stroma and/or by direct action on hematopoietic progenitors (77–79). Kennedy et al. revealed that BMAds induce myeloid-derived suppressor cells (MDSCs), particularly in mononuclear cells (CD11b^+^Ly6C^+^Ly6G^−^), which inhibit B lymphopoiesis by producing IL-1 (80). Additionally, BMAds can also activate inflammasomes, such as the nod-like receptor 3 (NLRP3), which directly inhibit B lymphopoiesis (40). Activation of inflammasomes can stimulate thymic degeneration (81, 82) and exert a negative effect on T-cell proliferation (83), likely contributing to systemic inflammatory conditions associated with advanced age.

There is growing evidence to support the involvement of chemokines such as C-motif chemokine ligand 2/monocyte chemoattractant protein 1 (CCL2/MCP-1) and cyclooxygenase-2 (COX-2) in regulation of the BM microenvironment (84). During inflammatory events, high expression of COX-2 is often coupled with CCL2/MCP-1 upregulation (85–87). The major COX-2 metabolite, prostaglandin E2 (PGE2), is known to induce differentiation of immunoregulatory cells like regulatory T-cells and MDSCs (41–44). Cox-2 inhibitors prevent CCL2/MCP-1 production by activated macrophages (88, 89). Under normal physiological conditions, COX-2 expression in macrophages is low but is increased in response to pro-inflammatory stimuli (90). In fact, the COX-2 expression and PGE2 release by macrophages were shown to be stimulated by CCL2/MCP-1 and to be important for macrophage migration (91–93). *In vitro* studies using conditioned media from BMAds demonstrated that macrophages are highly stimulated by BMAd-derived factors and that invasiveness increases with age (94). Obesity phenocopies aging with increased BMA, which has been shown to induce CCL2/MCP-1 and COX-2 within the BM (94), emphasizing a close relationship between immune response and BMA.

### Cellular Senescence

Aging studies have consistently shown a strong correlation between increased BMA and pro-inflammatory factors (18). It has been suggested that a sustained pro-inflammatory state may negatively impact the proliferative and differentiation capacities of surrounding cells. This effect is referred to as the “bystander effect” and most notably contributes to the accumulation of senescent cells in the BM, a process that naturally occurs with aging (46). Despite studies finding relatively low percentages (10–20%) of senescent cells in aged BMSCs, the bystander effect greatly impairs osteogenic capacities of non-senescent BMSCs, likely through senescence-associated secretory phenotype (SASP) factors (IL-1α, IL-1β, NF-κB, CXCL1/2, TGF-β, p21, p16, CCL2/MCP-1) and the resulting inflammation (95, 96).

BMAT expansion induces pro-inflammatory cytokines, which perpetuates the damaging effects on neighboring cells (46). In this pro-inflammatory microenvironment, BMSCs become senescent, resulting in decreased stem/progenitor cell number and decreased functionality (25, 47). In addition, the increased levels of pro-inflammatory cytokines promote ROS within the BM, further contributing to cellular senescence (23, 45). Flow cytometry analysis by Miggitsch et al. highlighted BMAds as a major contributor of ROS by determining higher ROS levels within femoral BMAds compared to subcutaneous white adipose tissue (WAT) from the thigh (45). Treatment of both tissues with ROS scavengers, N-acetylcysteine (NAC) and vitamin C, significantly reduced ROS levels within the BMAT compared to the WAT (45). The role of ROS in hematopoiesis has been well documented, thus these results demonstrate that BMAds limit the capacity of BMSCs to support the hematopoietic niche (97, 98).

Mimicking the potential effect of increased BMAT, Lo et al. showed that in conditions of elevated glucose *in vitro*, β-galactosidase activity and adipogenic differentiation markers (*Pparγ *and *Fas*) were notably increased while osteogeneic markers (*Runx2* and *Col1a1*) were decreased in BMSCs, indicative of altered differentiation potential (99). This hyperglycemic condition induces inflammation and senescence through oxidant-mediated autophagy, ultimately contributing to dysfunction of bone development and hematopoiesis in the BM microenvironment (100). BMP-2, an established pro-osteoblastogenic protein, can stimulate bone production in healthy, non-senescent BMSCs. However, in senescent cells recombinant BMP-2 upregulates pathways of inflammation, adipogenesis, and cell apoptosis (101). In mouse models, FOXP1, a regulator of the pro-adipogenic CEBPβ/δ complex in BMAT, has been shown to attenuate senescence through repressing p16^INK4A^ (encoded by *CDKN2A*), a cell cycle repressor that functions by inducing a G1 phase arrest (102). Collectively, BMAds play a critical role in inducing senescence of BMSCs, thereby determining the microenvironmental status in the BM compartment during aging.

## Potential Targets for Age-Related Bone Conditions

### Senolytics

Senolytics are a class of drugs that selectively induce apoptosis in senescent cells. Studies have shown reductions in age-related chronic inflammation led to functional restoration of bone regeneration through decreased senescence, increased stem/progenitor cell number, and increased osteogenic gene expression (25). In a pharmacological rescue experiment, Zhou et al. showed that BMSCs from aged mice (27 months old) had lower proliferation rates (30%) than young, 3-month-old mice (45%) (95). Twenty-four-hour treatment with dasatinib (generic chemotherapy; tyrosine kinase inhibitor) and quercetin (flavonol; antioxidant and chelating abilities) increased proliferation rates of the old BMSCs to 40% but did not affect the proliferative rates of the young BMSCs (95, 103, 104). Furthermore, dasatinib and quercetin treatments have been shown to improve osteogenic capacity in the aged BMSCs and reduce their expression of several senescence-related and inflammation markers including p21, p16^INK4A^, IL-6, CXCL1 and MCP-1 (95) in multiple aged tissues (105, 106). Therefore, clearance of senescent cells by senolytics shows promise in improving osteogenesis of aged BMSCs and ameliorating BM inflammation.

### miRNAs

In the past few decades, microRNAs (miRNAs) have emerged as key regulators of different aspects in development, homeostasis, and function. However, only a handful of miRNAs have been identified as capable of mediating adipocyte differentiation and function (107, 108). Multiple studies have implicated a potential role for miRNAs on post-transcriptional regulation of BMSC differentiation and aging (109, 110). For example, mice lacking miR-188, an age-associated miRNA found in the BM, showed substantial protection from bone loss and BMAT accumulation over time (109). In comparison, BMSCs transfected with miR-183-5p mimicked reduced cell proliferation and osteogenic differentiation and demonstrated increased cellular senescence (111). Therefore, miRNAs represent a unique class of therapeutic targets of bone inflammaging, given that their specific roles in the BM during aging become elucidated.

### Antioxidants

Given the positive effect of low-glucose conditions on senescent BMSCs, methods for glucose reduction have the potential to improve BM health through increasing mitochondrial respiration (99). Studies have shown that restricted glucose conditions increase the presence of antioxidant enzymes and decrease superoxide production, highlighting a therapeutic role for antioxidant defenses (99). An antioxidant and free radical scavenger, apocynin, was used to establish potential inhibition of cellular senescence, even in a senescence-accelerated mouse model, while concurrently improving osteogenesis (112). Similarly, treatment of aged rats with the aforementioned ROS scavenger, NAC, displayed an improved bone phenotype (113). Natural antioxidants have the potential to ameliorate concerns of age-related BMAT expansion. For example, phloretin, a flavonoid commonly found in apples, activates osteogenic gene OPG while promoting BMAd apoptosis to promote osteoblast differentiation, even in aged BMSCs (114). Given what we know about the link between inflammation and aging, it is not surprising to note that in addition to antioxidants, nonsteroidal anti-inflammatory drugs such as aspirin have been shown to counteract the effects of BMSCs senescence by improving cell proliferation and osteogenic differentiation (115, 116).

### Adipokines

Adipokines have the potential to regulate physiological functions including satiety, glucose homeostasis, energy expenditure (117), and inflammation (118). As a major regulator of the bone marrow niche with changes during inflammation and aging, adipokines are of great potential for future therapeutics. Numerous cytokines such as CCL2/MCP-1 (94), IL-6, and TNF-α (119) have elucidated roles in linking BMA with bone loss through inflammation. Of note, existing anti-TNF-α therapy infliximab and other TNF-α inhibitors have been shown to prevent age-related bone loss in various conditions (120, 121). In addition, pro-inflammatory adipokines shown to drive decline in bone health include leptin (31), resistin (32), chemerin (33), and adipsin (19). Among them, adipsin provides a straightforward relationship that might be of interest therapeutically by being produced abundantly in the BM and directly priming BMSCs. Furthermore, adipsin is involved in the alternative pathway (AP) of the complement system, a known activator of inflammation in the bone marrow further contributing to bone loss conditions (122–125). In human studies, patients with bone-related conditions such as post-menopausal bone loss and osteoarthritis displayed an increase in serum adipsin levels positively associated with other pro-inflammatory cytokines (126, 127). As such, current pharmacological advancements including the synthesis and pre-clinical characterization of adipsin inhibitors targeting the AP may be of interest in addressing inflammaging and bone loss (128).

## Conclusion

The development of BMAT is a normal physiological process and is arguably of importance in regulating BM microenvironment, skeletal homeostasis, hematopoiesis, endocrine function, and energy expenditure, and metabolism. However, extensive BMAT accumulation that occurs with aging and in clinical conditions such as obesity, calorie restriction/anorexia (20, 129), and in response to chemotherapy and irradiation treatments (130–132), suggests that aberrant BMAT formation has pathological implications. The increased adiposity within the BM exacerbates age-related inflammation and contributes to reduced bone health through physical changes in the bone matrix and defects in the BM stroma and HSCs. Ultimately, the age-associated shift of BMSCs toward adipogenesis promotes increased ROS, reduced HSC potential, dysfunctional immune cell response through increased myelopoiesis, and cellular senescence. As such, therapeutic interventions to maintain BMAT in appropriate quantity and quality may improve overall bone health, inflammaging, and senescence, further contributing to increases in life expectancy and quality of life for the elderly population.

## Author Contributions

NA and SC have contributed equally to this work and share first authorship. NA and SC wrote the manuscript. LQ and CR advised and edited the manuscript. All authors contributed to manuscript revision and approved the submitted version.

## Funding

Funding was supplied by the NIH/NIDDK R24 DK092759-06 (CR), NIH U19AG060917 (CR), NIH/NIDDK R01 DK112943-05 (LQ), R01 DK128848 (LQ), and NIH/NIDDK F31 DK124926 (NA).

## Conflict of Interest

The authors declare that the research was conducted in the absence of any commercial or financial relationships that could be construed as a potential conflict of interest.

## Publisher’s Note

All claims expressed in this article are solely those of the authors and do not necessarily represent those of their affiliated organizations, or those of the publisher, the editors and the reviewers. Any product that may be evaluated in this article, or claim that may be made by its manufacturer, is not guaranteed or endorsed by the publisher.
